# How can entrepreneurial interventions in a university context impact the entrepreneurial intention of their students?

**DOI:** 10.1007/s41959-022-00083-x

**Published:** 2022-12-26

**Authors:** David Bozward, Matthew Rogers-Draycott, Cynthia Angba, Carol Zhang, Hongyu Ma, Fulin An, Federico Topolansky, Luca Sabia, Robin Bell, Emily Beaumont

**Affiliations:** 1 The Business School, University of Gloucestershire, Oxstalls Lane, Longlevens, Gloucester, Gloucestershire, GL2 9HW UK; 2 Warwick University, Coventry, UK; 3 Royal Agricultural University, Cirencester, UK; 4 College of Economics and Management, Northwest A&F University, Shaan Xi Province, Yangling, China; 5 International Centre for Transformational Entrepreneurship, Coventry University, Coventry, UK; 6 Worcester Business School, University of Worcester, Worcester, UK

**Keywords:** Entrepreneurial intentions, Entrepreneurial interventions, Entrepreneurship education, Enterprise

## Abstract

This paper explores the link between the entrepreneurial intention of students in higher education and the entrepreneurial interventions an institution can provide to support them. The study uses data collected from 679 undergraduate students from Chinese and UK Universities. The instrument for data collection was a paper-based questionnaire. This study uses the integrated model of entrepreneurial intentions as the theoretical underpinning for this approach. The initial findings highlight the perceived need for a range of entrepreneurship interventions, with business training programmes being the highest priority, followed by mentoring, specialist business advice, low-cost finance, business networking events and enterprise clubs. It also shows that those with different Intention Horizons do request a different portfolio of interventions. The paper provides an evidence-based approach to entrepreneurship education design and the development of interventions to support a range of students with and without entrepreneurial intention. This work suggests a previously under-articulated relationship between the nascent entrepreneur’s Intention Horizon, university interventions, and entrepreneurial action. There are numerous calls for further contextualisation of entrepreneurship education which this paper fulfils (Baron and Shane in Psychol Entrepreneurship 19-39, 2007; Byrne et al. in Edward Elgar Publishing, 2014). It further develops the narrative around both contextualisation, the previous experience of the students and the range and importance of these interventions to support the creation of a new venture.

## Introduction

The processes and drivers that lead to venturing are still regularly debated across the entrepreneurial research literature (Mirjana et al., 2018). These conversations have led some to conclude that behaviour (as a catalyst for action) is central to unpicking this complex issue and, because intention can be considered to be the foundation of behaviour, many researchers (Tornikoski & Maalaoui, 2019) have chosen to use Ajzen’s (1991) Theory of Planned Behaviour (TPB) to explore the ways in which intention might predict behaviour, and by extension, venturing.

In the context of entrepreneurial studies, TPB is most often used to explore the ways in which attitudes, norms and perceived behavioural controls propel nascent entrepreneurs toward venturing. To date, studies have tended to focus on testing TPB to explore the robustness of the link between intention and action in a range of contexts (Kautonen et al., 2013). The problem with this literature is that it largely ignores the complex relationships and interventions which may have helped to foster the development of intention (Bae et al., 2014). Several studies have shown that entrepreneurship education has an affirmative impact on intention (Boahemaah et al., 2020; Bozward et al., 2022; Shiri et al., 2017; Zampetakis et al., 2013), but none have connected intent to specific interventions.

One reason for this may be that exploring particular interventions in a meaningful fashion necessitates a consideration of a temporal factor, as it is recognised that individuals may act on intention differently over time (Nasar et al., 2019). TPB, as Ajzen himself admits, finds it difficult to address this relationship (Tornikoski & Maalaoui, 2019). A small number of researchers have begun to consider how intention changes over time (Boissin et al., 2017; Hallam et al., 2016; Nasar et al., 2019; Reitan, 1996; Zhang et al., 2018); however, as this paper will show, there is debate as to the impact of interventions on intention over time which new insights can contribute to.

Given this reading, the authors contend that the relationship between the nascent entrepreneur’s attitudinal characteristics, their intention (over time), and the impact on this of specific interventions is an under-researched area of scholarship. Our question, therefore, is how can institutions nurture attitudes, characteristics and intent in their student populations, over time, and through education-based interventions, thus supporting the transition to action.

To address this, the paper focuses on intent, specifically the relationship between intent and interventions in an education context, to better understand what institutions can do to foster intent and translate this into action for this group of students. In doing so the paper builds on several key sources: Najafabadi, Zamani and Mirdamadi (2016), Boissin et al. (2017), Zhang et al. (2018) and Nasar et al. (2019) to explore intent over time, and Preedy and Jones (2015) to understand specific interventions. Through these papers, the authors will evolve a model for intent, link this to interventions, and seek to understand how this relates to entrepreneurial activity.

## Intention

Entrepreneurial intentions by an individual can be defined as a self-acknowledged view that they intend to create a new business venture and intentionally plan to do so at some point in the future (Thompson, 2009).

In Najafabadi, Zamani and Mirdamadi (2016), the author’s identify two models for entrepreneurial intention, the theory of planned behaviour (TPB) (Ajzen, 1991; Bird, 1988; Boyd & Vozikis, 1994), and Shapero’s model of the entrepreneurial event (SEE) (Shapero & Sokol, 1982). TPB suggests that behaviour is driven by intention which is in turn motivated by attitude, subjective norms, and perceived behavioural control. SEE models the intention event as being driven by perceived desirability, propensity to act and perceived feasibility.

**Fig. 1:**
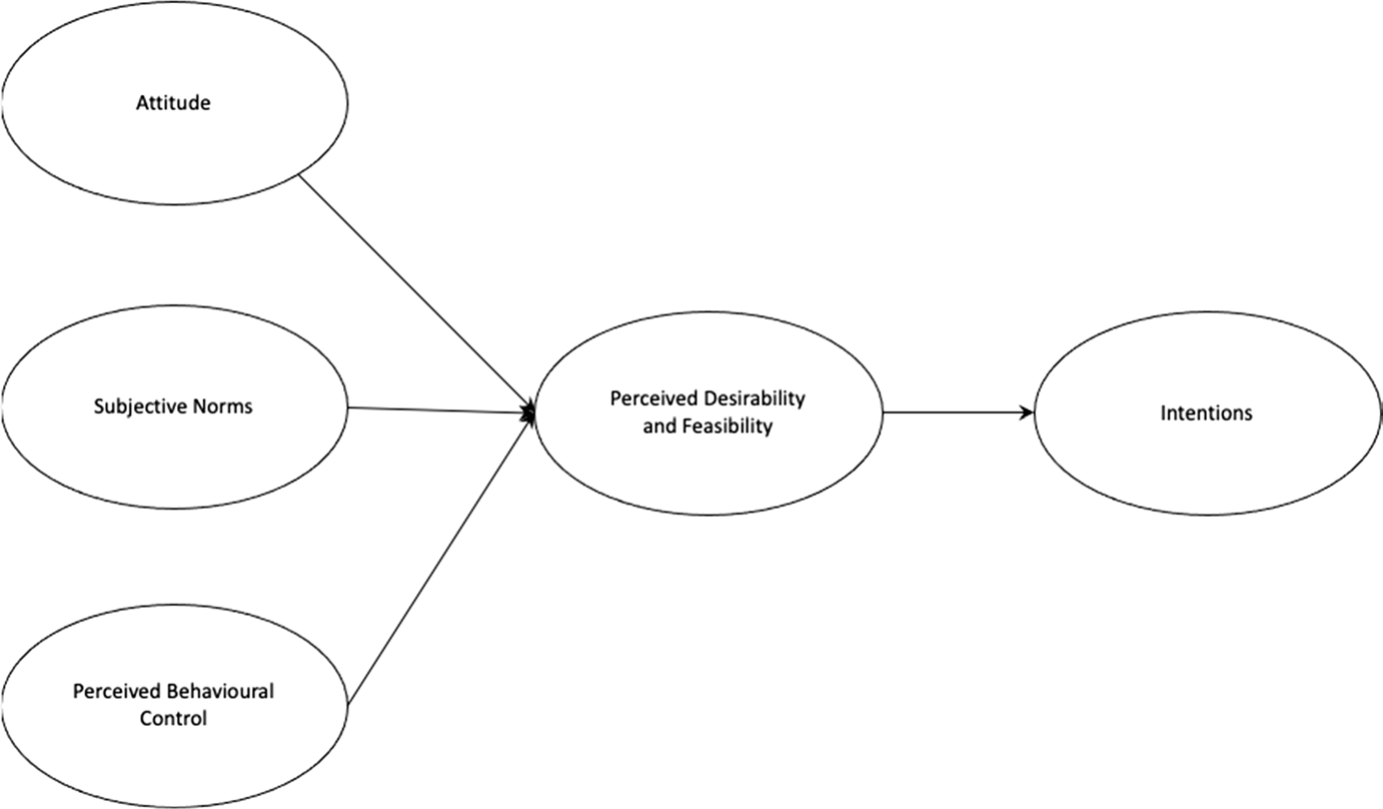
An integrated model of entrepreneurial intentions (Source: Iakovleva & Kolvereid, 2009 p74)

These models have been critically reviewed by numerous authors, most notably by Krueger et al. (2000), but it was Iakovleva and Kolvereid (2009) who first brought them together, concluding that intention is driven by perceived desirability and feasibility which is, in turn, motivated by attitude, subjective norms, and perceived behavioural control (Fig. 1).

In an entrepreneurial context, this means that a person’s attitudes, beliefs, upbringing, values, and their awareness of the ease or difficulty of the execution of the behaviour of interest will all inform whether they are attracted to act entrepreneurially in a given context, and this will affect their intention to do so.

**Fig. 2:**
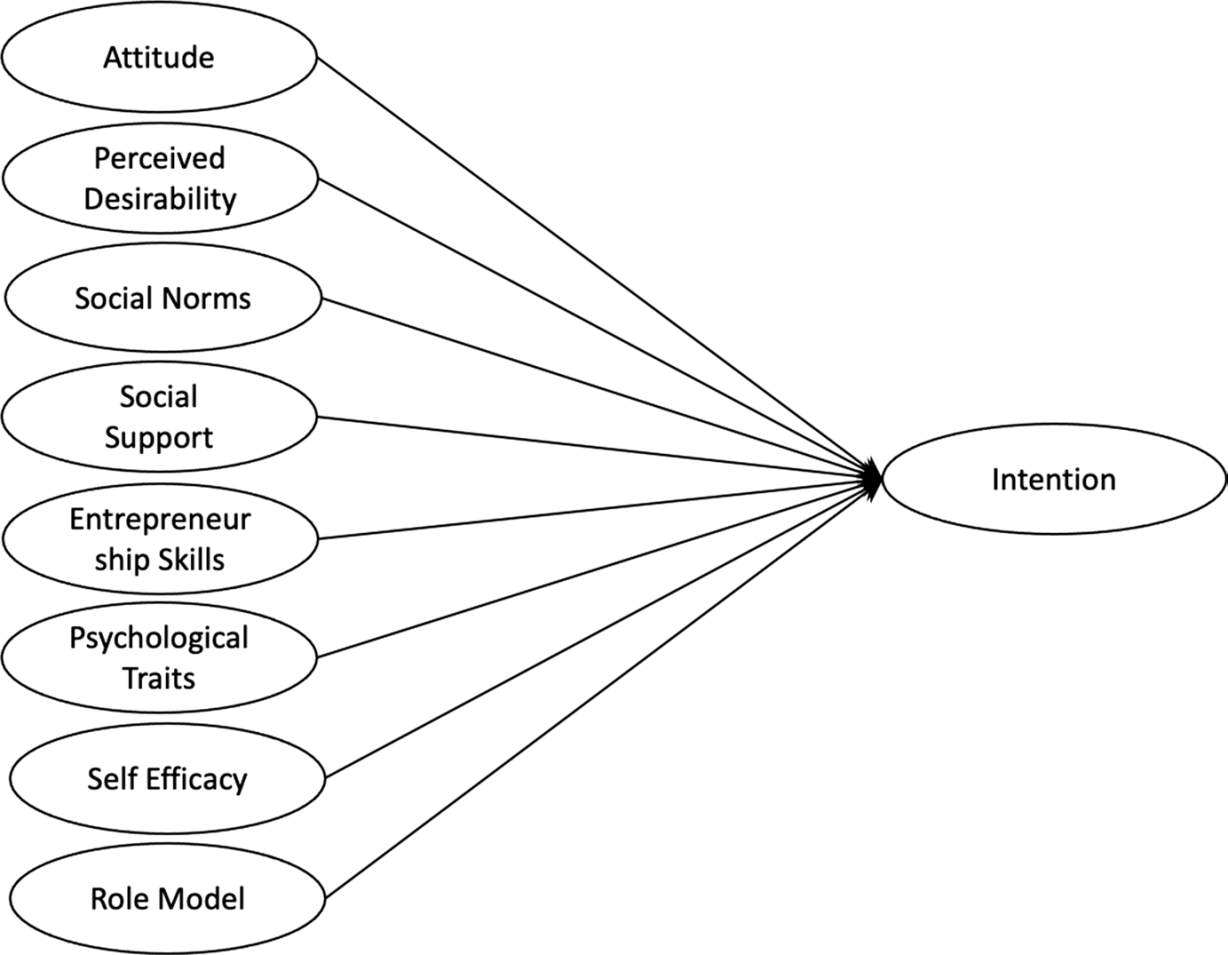
Model for entrepreneurial intentions of agricultural students (Source: Najafabadi et al., 2016 p6)

Recently, Najafabadi, Zamani and Mirdamadi (2016) have proposed a more granular model of the factors driving entrepreneurial intent of agricultural students. This includes some of the features of Iakovleva and Kolvereid’s model along with several additions and reconfigurations (Fig. 2).

In this model, the author’s showed that established entrepreneurial skills had the greatest bearing on intention, followed by self-efficacy, attitude, physiological characteristics and social norms, all of which they note are well-established points in the wider literature. The primacy of existing entrepreneurial skills is interesting, although ultimately unsurprising given the growing consensus that prior knowledge and experience play in underpinning the process of entrepreneurship through the mechanism of judgement (Gieure et al., 2020; Rogers-Draycott, 2021) which has also been shown in agricultural students specifically (Abdullah & Samah, 2014). Perhaps the most compelling insight in the paper is the negative effect of role models on intention, which the author’s themselves note contradicts much of the established literature, especially that which is based on the Global Entrepreneurship Monitor (GEM, 2022). The author’s consider that this might be a result of a lack of skills and experience on the part of the nascent entrepreneur. In practice, they suggest that the lack of skills and experience means that the individual does not have sufficient ability to critically judge the advice, or actions, of the role model resulting in the disincentive effect they have measured.

**Fig. 3:**
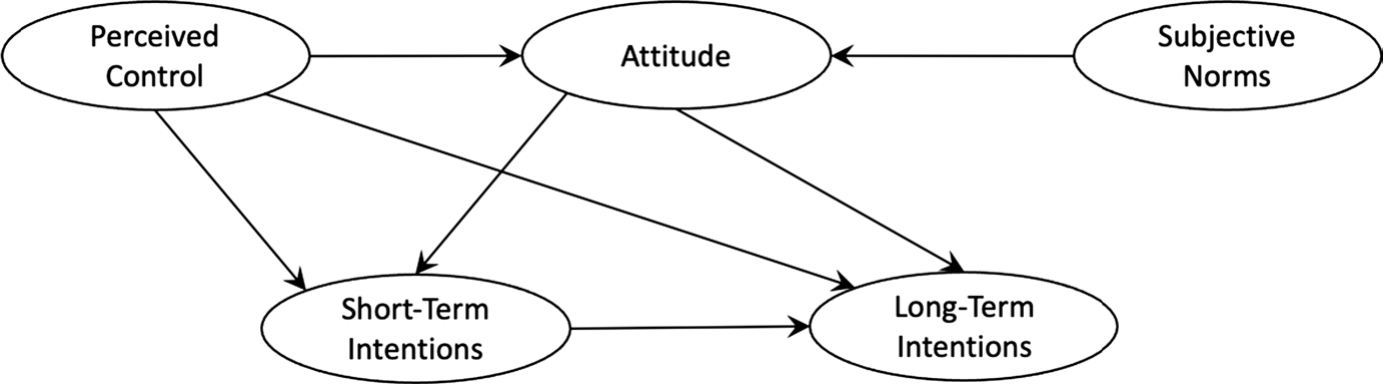
Internal submodel of entrepreneurial intention (source: Bossin et al., 2017 p29)

Boissin et al. (2017) also explore the determinants of entrepreneurial intention, with a focus on impacts. Therein, the author’s apply a modified version of Iakovleva and Kolvereid’s model although, again, they do not agree that the initial factors inform a perception that then affects intention (Fig. 3).

While this is useful as part of the overarching schema, it is not the central contribution this paper makes to our understanding of intention, that is their conception of intention itself. Boissin, Favre-Bonté and Fine-Falcy show that intention is not a binary variable in which the nascent entrepreneur either will start a business today or will not, instead, they explore the notion of short- vs. long-term intention. Their work demonstrates that, generally, intention to act entrepreneurially is greater in the long term, the reasons for this are complex, but it is likely underpinned by a belief that a students’ skills, experience, and lifestyle might be more aligned to entrepreneurial action once they have more stability and knowledge later in life.

Other notable perspectives on the relationship between intention and time include: Reitan (1996), who found evidence suggesting that situational variables exercise more influence on short-term intentions than on long-term intentions; Zhang et al. (2018) who showed that, generally, entrepreneurial experience has a greater impact on long-term intention. However, for those with a social motive, the impact was greater in the short term. They also found that Chinese university students have less long-term intention than their US counterparts; and, Nasar et al. (2019) who used a near future and a distant future intent model. They concluded that strong short-term entrepreneurial intention reduces long-term entrepreneurial intention.

Taken together these sources present a confusing picture of the relationship between time, intention, and interventions. The work of Reitan (1996) and Nassar et al. (2019) would seem to suggest that interventions will most likely impact short-term intention, and that this will reduce intention in the long term. While the work of Boissin et al. (2017) and Zhang et al. (2018) asserts that intention to act entrepreneurially is greater in the long term, likely the result of experience or perception, with the caveat that motive and/or culture may impact this.

**Fig. 4:**
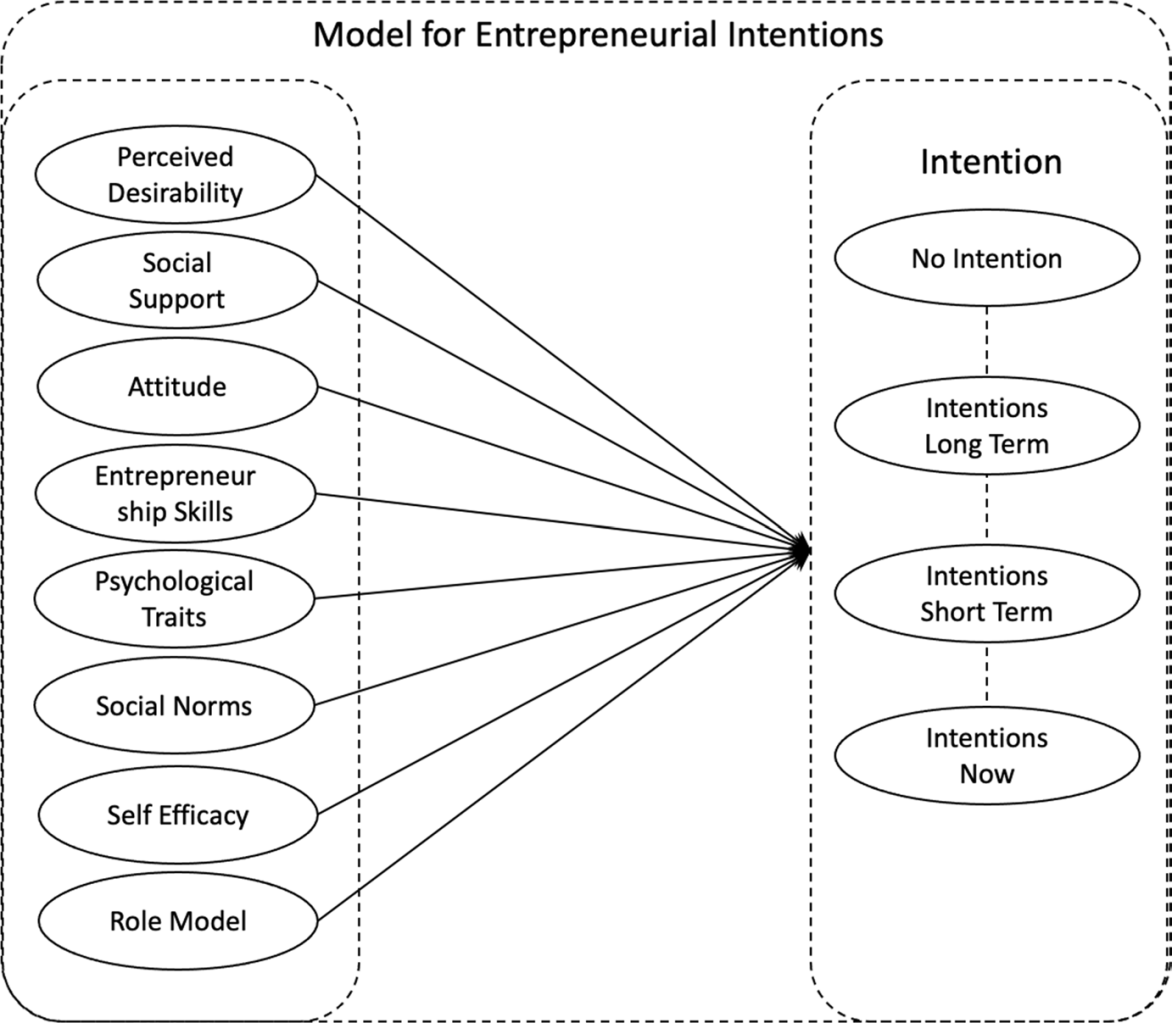
Entrepreneurship intention model

Our work combines ideas from Najafabadi, Zamani and Mirdamadi (2016) and Boissin et al. (2017) into a single model of entrepreneurial intention, and links this to time horizons which are inspired by Reitan (1996), Boissin et al. (2017), Zhang et al. (2018), and Nasar et al. (2019) (Fig. 4.).

In this model, we conceptualise a range of established factors drawn from the literature as the forces underpinning intention. In an effort to provide new insights into the debate surrounding intention, time, and interventions, we propose a move beyond the exploration of short- vs. long-term interventions which has been the focus of prior work. Instead, we suggest four distinct Intention Horizons:

- No Intention
- Long-term Intention—two years or more
- Short-term Intention—in six months' time
- Intentions Now—over the last twelve months or currently developing a business idea

It is our contention that this increased granularity should provide deeper insights into the ways in which interventions affect intention over time, which might offer an opportunity to resolve some of the confusion this paper has already highlighted.

## Entrepreneurship interventions

There are a range of educational interventions which can be applied to support entrepreneurial intention and successful venturing. Preedy and Jones, (2015) explored extra curricula interventions in 20 universities in the UK with a focus on their offerings. They found that, on average, that universities offered 10 of the 18 interventions with business networking events, enterprise bootcamps and training workshops, and business advice sessions being the most popular. Preedy and Jones, (2015) stated that “it is not expected that all the participants would be aware or able to recall all enterprise support activities ongoing at their university and consequently there may be under representation of activity”. Therefore to facilitate comparisons of their effect on intention, we selected 6 interventions, that happen to be among the most common, which were offered across the institutions.

### Business training programme

The need for entrepreneurship education has been well established (Hägg & Gabrielsson, 2019; Matlay, 2008) as a means of developing human capital (Martin et al., 2013) through this form of intervention. The findings also show that these in-curricula programmes have a noteworthy effect on the students’ abilities to generate a greater number and more innovative business ideas. It has also been shown to increase entrepreneurial intention (Zhang et al., 2014).

### Mentoring

The role of a mentor in both industry and education is established and proven to support business growth and entrepreneurial intention (Galvão et al., 2020; Ting, Feng and Qin 2017). It has also been reported to be beneficial in terms of skills and competence development to a number of industries (Eissner et al., 2018; Morshedi Estahbanaty, 2014; Mwaura, 2012) and locations (Ferreira et al., 2020; Kyrgidou & Petridou, 2013; Sawatsky et al., 2016).

### Specialist business advice

Businesses have access to a range of specialist business advice (Bennett & Robson, 1999), from accountants and lawyer to government sponsored advisors and sector specialists. For example, in farming, McElwee, 2006 identified the need for business advice which is both industrial in its depth and financial accuracy in its breath is vital for the success of these new ventures. Fitz-Koch et al. (2018) supports this in calling for further development in sector context entrepreneurship.

### Low-cost finance

SAXENA, (2012) in India and Nukpezah et al., (2017) in Ghana to Aisaiti et al. (2019) in China have all highlighted the need for low-cost finance in the emerging markets of the world. This issue stems from a need for financial awareness and literacy (Akoto et al., 2017; Gaurav & Singh, 2012; Liu et al., 2021) on one side to available financial institutions on the other (which is outside the scope of this research paper).

### Business networking events

Networking has been shown to be beneficial on a number of levels for both the entrepreneur and their new venture (Johannisson, 2009) and is shown to provide a significant relationship between networking and productivity (Marritz, 2010). For student entrepreneurs, networking has been shown to also develop tacit knowledge (McAdam et al., 2008) and facilitate faster commercialisation (Pittaway, et al., 2004).

### Pre-Start enterprise clubs

The role of student led clubs has been shown to develop a range of benefits for nascent entrepreneurial students from learning by doing (Pittaway et al., 2015) to experiential learning opportunities, increased awareness of enterprise support, networking skills development and leadership competence development (Preedy & Jones, 2017) and organisational development, management and performance measurements (Bozward, Penwarden and Depinay 2013). Sansone et al. (2021) identified the connection between students within entrepreneurial students-led organisations and the intention to start a new business.

## Research model and questions

In the development of the theoretical framing of this paper, we have proposed a link between entrepreneurial intention and institutional interventions; therefore, we propose three research hypotheses to investigate this link:

### H1

Those with intention have a greater perceived requirement for entrepreneurial interventions than those with no intention.

### H2

Those with near term intention will have a greater requirement for interventions to those with longer-term intention.

### H3

The portfolio of interventions required is determined by the intention term.

**Fig. 5:**
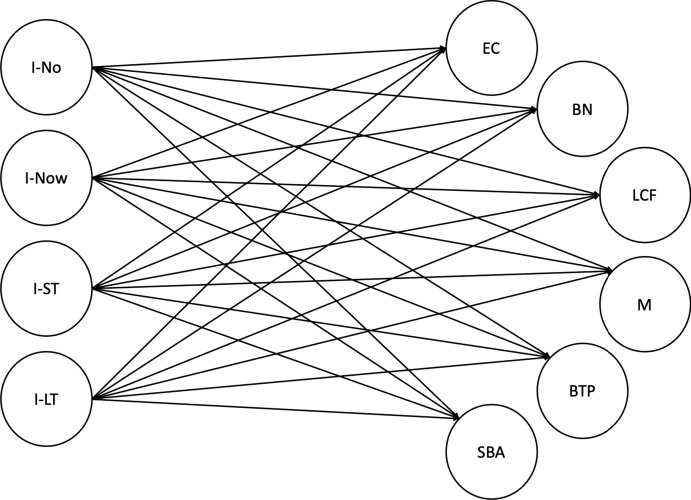
Intention intervention research model

We conceptualise a relationship between four Intention Horizons: No Intention (I-no), Intention Now (I-Now), Intention Short Term (I-ST), and Intention Long Term (I-LT) and six institutional interventions: Business Training Programme (BTP), Mentoring (M), Specialist Business Advice (SBA), Low Costs Finance (LCF), Business Networking Events (BN), and Pre-Start Clubs (EC) as shown in Fig. 5.

## Research method

The English language GEM survey was translated into Chinese. The translated survey was then tested to ensure the questions were “functionally equivalent for the purposes of analysis” (Scheuch, 1968 p 113-4). This means that the responses to questions should represent the same concepts we want to measure across these multicultural and multinational groups (Harkness, Villar and Edwards 2010).

The quantitative data were gathered from students through a self-administered anonymous paper-based GEM survey in the language of the tuition of that university, and the following six questions are related to this research:

### Intention Horizon questions

- ● Now Started
  - ○ You are, alone or with others, currently trying to start a new business, including any type of self-employment, or selling any goods or services to others
  - ○ You are, alone or with others, currently trying to start a new business or a new venture for your employer—an effort that is part of your normal work
  - ○ Over the past twelve months have you done anything to help start a new business, such as looking for equipment or a location, organising a start-up team, working on a business plan, beginning to save money, or any other activity that would help launch a business?
- ● Short Term
  - ○ Have you recently thought about starting your own business, buying into an existing business, or becoming self-employed?
  - ○ I n the next six months will there be good opportunities for starting a business in the area where you live?
- ● Long Term
  - ○ As a long-term option, would you prefer to run your own business or be employed by others?
  - ○ Are you, alone or with others, expecting to start a new business, including any type of self-employment, within the next three years?
  - ○ Is starting a business something, you are thinking of doing within the next two years or so, or further in the future than that?
- ● No Intention
  - ○ Those who provided a negative indication to all the above questions.
    Interventions question
- ● Which of the following would help you to start a business…? (Business Training Programme, Pre-Start Enterprise Clubs, Business Networking Events, Low-cost finance, Mentoring, Specialist business advice)

The data were collected from four universities, three in China and one in the UK, all of which specialise in agricultural higher education. They were the Henan Agricultural University (HAU), based in Zhengzhou, Henan province, China; the Northwest Agriculture and Forestry University (NAFU), based in Xianyang, the Shaanxi province, China; Shandong Agricultural University (SDAU), based in Shandong province, China; and Royal Agricultural University (RAU), based in Gloucestershire, UK. The data were collected over three academic years, 2018, 2019 and 2020 before COVID-19 restriction took place.

The survey was completed by 679 (second year in China and first year in UK) Bachelors Undergraduate students, studying on business and agricultural programmes, 201 from HAU, 162 from NAFY, 197 from SDAU and 119 from RAU. The average age of the students was 20.9 years old (HUA 19.9, NAFY 21.4, SDAU 20.2 and RAU 20.9 years old). All students except 2 were in the age range from 18 to 24. Across this student group, 62% of students were female, with HAU having 60%, NAFY having 51%, SDAU having 75% and RAU having 61% female student’s respondents.

## Results

For each intervention, we addressed each of the Intention Horizons identified, e.g. No Intention, Intention Long Term, Intention Short Term and Intention Now. The table below provides the percentage of those who selected the interventions. Students were allowed to select more than one intervention (Table 1).

**Table 1:** Selected interventions by experience and intention

	Business training programme (%)	Mentoring (%)	Specialist business advice (%)	Low costs finance (%)	Business networking events (%)	Pre start clubs (%)	Total in group (N)
No intention	27	24	17	20	20	15	221
intention now	43	40	37	40	20	21	247
Intention short term	53	43	45	32	31	28	307
Intention long term	67	53	48	41	35	27	261

Those with No Intention selected on average 1.23 interventions, whilst those who had an Intention Now selected 2.01, those with Short-Term Intention, 2.32, and those with Long-Term Intention 2.73 interventions. This demonstrates that those with a longer-term view of entrepreneurships are open to more interventions.

### Business training programme

74% (504/679) of all students selected this intervention making it the intervention of choice for those with and without intention. It is interesting to see that the largest percentage is from those who see entrepreneurship as a long-term ambition, with 67% selecting it. The second highest is those who have short-term ambition of starting a business, with 53% selecting this intervention. Meanwhile, only 43% of participants with Intention Now selected business training which suggests that the majority of these students consider they have the business skills and networks to start.

### Mentoring

This was selected by the second highest number of all students with 422, or 62% of students selecting this intervention. Those with a long-term ambition have the highest (53%) selection of this intervention, with those who intend to start a business in the short term as second highest (43%).

### Specialist business advice

This intervention came third with 393 (58%) of all (679) students selecting it. The data in this intervention follow those of other interventions highlighted above.

### Low-cost finance

Whilst those with long-term ambitions selected this intervention more than any other with 41%, Intervention Now selected it 40%, indicating that it may have a higher demand for current nascent entrepreneurs.

### Business networking events

Achieved only 41% (280/679) of all students selecting this intervention. Intention Now and those who have no intention have the same percentage with 20%, indicating this has a wider benefit to those just looking to start a business. Those with intentions in the short and long term (31% and 35%) indicate that the longer term the intention the greater the perceived need.

### Pre-start enterprise clubs

The intervention secured the least students with 36% (243/679) overall. Interestingly, the short-term intention group was the highest with 28%.

The next stage was to understand how these interventions are grouped within the students' perception. This was done by conducting a spearman’s rho correlation of the interventions as shown in Table 2.

**Table 2:** Correlation between education interventions

	Intervention	M	S.D	1	2	3	4	5	6
1	Business training programme	0.43	0.496	1.00					
2	Pre-start enterprise clubs	0.22	0.412	0.464**	1.00				
3	Business networking events	0.26	0.439	0.541**	0.603**	1.00			
4	Low-cost finance	0.28	0.451	0.560**	0.551**	0.711**	1.00		
5	Mentoring	0.37	0.483	0.695**	0.422**	0.538**	0.551**	1.00	
6	Specialist business advice	0.32	0.466	0.558**	0.447**	0.527**	0.512**	0.731**	1.00

This analysis shows that the highest significant correlations exist between Mentoring and Specialist Business Advice (0.731), Business Networking and Low Costs Finance (0.711) and then Business Training and Mentoring (0.695). The lowest correlations exist between Clubs and Business Training Programmes (0.464), Clubs and Specialist business advice (0.447) and Clubs and Mentoring (0.422); however, these are still highly correlated.

The next stage was to understand the correlation relationships between the intervention and the types of intention which is shown in Table 3. It should be viewed considering the percentages already presented in Table 1.

**Table 3:** Correlation between intention timescale and intervention required

	Business training programme	Mentoring	Specialist business advice	Low-cost finance	Business networking events	Pre-Start enterprise clubs
No intention	− 0.233**	− 0.185**	− 0.225**	− 0.122**	− 0.095*	− 0.106**
Intention now	− 0.018	0.004	0.054	− 0.107**	− 0.119**	− 0.022
Intention short term	0.205**	0.128**	0.273**	0.107**	0.101**	0.138**
Intention long term	0.385**	0.219**	0.290**	0.192**	0.137**	0.096*

** = correlation is significant at 0.01 level

* at a 0.05 level

There is a clear negative correlation between those with No Intention and the interventions. This should be expected as those students who do not intend to start a business could be seen to have negative interest. The only intervention which did not gain a significant correlation was Business Networking which appears to be just as important for all groups; however, it was still negatively correlated.

For Intention Now, there is a significant negative correlation with Low Costs Finance which will require further investigation. There is also a significant correlation with Business Networking demonstrating that those who have started a business, may have secured the other interventions, but are still open to further discussions.

For Short Term, all interventions were significantly correlated, with Specialist Business Advice highest, then Business Training and Enterprise Clubs.

For Long Term, all interventions except clubs were significantly correlated, with Business Training the highest, followed by Specialist Business Advice and Mentoring.

The correlations present a similar picture to those within the descriptive statistics presented in Table 1; however, these relationships indicate the portfolios of interventions which would be acceptable to those with certain types of intervention.

## Regression analysis

A series of logistic regressions were then conducted to assess the effect of each of the types of intervention on each of the types of Intention Horizon and is summarised in Table 4.

**Table 4:** Regression analysis of interventions and intentions

		B	S.E	Wald	df	Sig	Exp(B)	95% C.I. for EXP(B)	
								Lower	Upper
Business training programme	Intention = No	− 0.631	0.247	6.549	1	0.010*	0.532	0.328	0.863
	Intention now	− 0.546	0.107	25.926	1	0.000**	0.579	0.469	0.715
	Intention short term	0.221	0.162	1.863	1	0.172	1.248	0.908	1.715
	Intention long term	0.892	0.129	47.846	1	0.000**	2.440	1.895	3.142
	Constant	− 0.379	0.194	3.794	1	0.051	0.685		
Mentoring	Intention = No	− 0.935	0.243	14.827	1	0.000**	0.392	0.244	0.632
	Intention now	− 0.409	0.101	16.416	1	0.000**	0.665	0.545	0.810
	Intention short term	0.042	0.152	0.076	1	0.783	1.043	0.774	1.405
	Intention long term	0.296	0.113	6.863	1	0.009**	1.344	1.077	1.677
	Constant	− 0.218	0.185	1.395	1	0.238	0.804		
Specialist business advice	Intention = No	− 0.552	0.267	4.283	1	0.038*	0.576	0.341	0.971
	Intention now	− 0.308	0.103	8.911	1	0.003**	0.735	0.600	0.900
	Intention short term	0.539	0.160	11.401	1	0.001**	1.714	1.254	2.343
	Intention long term	0.484	0.117	17.244	1	0.000**	1.623	1.291	2.040
	Constant	− 1.052	0.197	28.619	1	0.000**	0.349		
Low-cost finance	Intention = No	− 0.613	0.260	5.582	1	0.018*	0.542	0.326	0.901
	Intention now	− 0.538	0.116	21.610	1	0.000**	0.584	0.466	0.733
	Intention short term	0.182	0.162	1.259	1	0.262	1.199	0.873	1.646
	Intention long term	0.317	0.120	6.970	1	0.008**	1.373	1.085	1.736
	Constant	− 0.751	0.199	14.286	1	0.000**	0.472		
Business networking events	Intention = No	− 0.639	0.265	5.815	1	0.016*	0.528	0.314	0.887
	Intention now	− 0.593	0.124	23.023	1	0.000**	0.553	0.434	0.704
	Intention short term	0.146	0.167	0.763	1	0.382	1.157	0.834	1.603
	Intention long term	0.211	0.124	2.913	1	0.088	1.235	0.969	1.574
	Constant	− 0.753	0.205	13.520	1	0.000**	0.471		
Pre-start clubs	Intention = No	− 0.467	0.285	2.684	1	0.101	0.627	0.359	1.096
	Intention now	− 0.296	0.117	6.400	1	0.011*	0.744	0.592	0.936
	Intention short term	0.374	0.173	4.663	1	0.031*	1.454	1.035	2.042
	Intention long term	0.059	0.127	0.216	1	0.642	1.061	0.828	1.359
	Constant	− 1.238	0.215	33.090	1	0.000**	0.290		

** = correlation is significant at 0.01 level

* at a 0.05 level

No Intention has a significant relationship with Business Training Programmes and Mentoring, with Pre-start Clubs (0.0101), Business Networking Events, Low Cost Finance and Specialist Business Advice also demonstrating notable, but less significant relationships.

Short-Term Intention has only one significant relationship and that is with the dependent variable Specialist Business Advice.

Business Training Programmes and Mentoring have a relationship between dependent variables: No Intention, Intention Now, and Long-Term Intention. For Long-Term Intention, the confidence interval is entirely above 1.0, which means that exposure to this predictor increases the odds of the outcome. For No Intention and Intention Now, the adjusted odds ratio is below 1.0 and the confidence interval is entirely below 1.0, as such, exposure to these predictors decreases the odds of the outcome. The regression suggests that those who have a long-term intention may gain the most from programmes, but that these will also have an impact on individuals with other Intention Horizons.

Specialist Business Advice has significant relationships with Long Term, Short Term, Intention Now, and No Intention, suggesting that most Intention Horizons would benefit from this intervention. The significant relationship with No Intention is compelling and merits further exploration, perhaps it suggests that specialist advice can help to inspire those with No Intention by providing detailed domain insights.

Low-cost Finance has a significant relationship with Intention Now; we contend that this is likely because these students will need access to finance most immediately to start their ventures. The adjusted odds ratio is below 1.0 and the confidence interval is entirely below 1.0; therefore, exposure to the predictor decreases the odds of the outcome. There are also relationships to Long-Term Intention and No Intention. This suggests that access to finance is important for most groups, the reasons for this are not specified, but we speculate that finance might motivate individuals toward action by reducing the inherent risk of utilising personal or familial capital.

Business Networking Events also has a significant relationship with Intention Now; this is likely the result of these students wanting to share their venture and seek additional support. There is also a significant relationship with No Intention which requires further investigation as the reasons for this are not immediately clear. The adjusted odds ratio for both of these are below 1.0, and the confidence interval is entirely below 1.0; therefore, exposure to the predictor decreases the odds of the outcome.

Pre-Start Enterprise Clubs have a relationship with Intention Now and Short-Term Intention. For both of these, the adjusted odds ratio is above 1.0 and the confidence interval is entirely above 1.0; therefore, the exposure to either of these predictors increases the odds of the outcome. We suggest that access to peer networking and support may be particularly important for students who are close to venturing as mechanisms to help them shape their ideas, and refine their venture.

## Discussion

This research, which was designed to investigate the relationship between entrepreneurial intention and interventions, posed three hypotheses which will now be considered in the light of the findings.

### H1

Those with Intention have a greater perceived requirement for entrepreneurial interventions than those with no intention.

The results in Table 1 highlighted the greater perceived requirement (value) of entrepreneurial interventions in those with intentions to start a business at some stage. Indeed, those with No Intention selected on average 1.23 interventions, whilst those who had an Intention Now selected 2.01, those with short-term ambition 2.32, and those with long-term ambition 2.73 interventions. In this study, those with No Intention almost always selected lower percentages of interventions.

In addition, there is a mostly negative significant correlation between those with No Intentions and the investigated interventions (Table 3). This might be expected as students who do not intend starting a business may be less interested. The only intervention which did not significantly correlate was that of Business Networking, although it still correlated negatively. This may echo the phenomena observed by Najafabadi, Zamani and Mirdamadi (2016) in relation to role models, and may result from the same lack of skill and experience in those with No Intention. Furthermore, from Table 4, those with No Intention only had a significant relationship with Mentoring, and less so with Networking Events and Lost Cost Finance. Whilst those with Intention Now have a significant relationship to all except Pre-start Clubs, those with Long-Term Intention see significance in Business Training, Mentoring, and Finance although not with Networking and Pre-start Clubs.

As a result, this hypothesis was confirmed.

### H2

Those with near term intention will have a greater requirement for interventions to those with longer-term intention.

In general, those with longer-term intentions selected more interventions in Table 1. Furthermore, the longer the intention term the higher (on average) the correlation in Table 3. Finally, the significance is lower (short term compared to long term) which shows a stronger relationship.

Those with a longer-term view of entrepreneurship were open to more interventions, perhaps because they were less confident in knowing what they might need in the future, had less knowledge, or simply wanted to acquire as much know-how as possible. Those with Intention Now, or intention over the short term, might have already begun to gather information and made enquiries, or early networks. Those with short-term ambitions only have a significant relationship with Specialist Business Advice. This requires further research but suggests this may be seen to be the most valuable knowledge to them in their particular situations.

This hypothesis was rejected.

### H3

The portfolio of interventions required is determined by the intention term.

Drawing on the information in Table 1, it is possible to show which interventions are considered most important for each group of Intention Horizons:

- No Intention cohort: Business Training Programme;
- Intention Now cohort: Business Training Programme, Mentoring, Low-cost Finance, and Specialist Business Advice;
- Short-Term Intention cohort: Specialist Business Advice; and
- Long-Term Intention cohort: Business Training Programme.

The data in Table 2 indicated that the highest correlations between the interventions were seen between Mentoring and Specialist Business Advice (0.731); Mentoring and Specialist Business Advice (0.731); and Business Training and Mentoring (0.695), indicating that students who chose one were more likely to choose the other.

While this indicates that some interventions are important to most groups of Intention Horizon, it does show that particular interventions (or combinations) may be more important to some, as such we consider this proved, although we accept that further research could lead to better insights herein.

This hypothesis was confirmed.

Taken together, this suggests that those students with intentions of starting a business, in general, have a greater requirement for entrepreneurial interventions than those who may not be interested. Interventions will vary depending on the specific circumstances of the student and the timescale of their ambition. Those with a longer-term vision should be viewed as more open to more interventions than those with a shorter-term vision. This may be a result of the former wanting to accumulate as much knowledge as possible, not having a plan in place, or the latter having started the journey and looking for specific direction and more expert knowledge. It should also be noted that the impact of these interventions may not be in the short term and nor should they expect the student to act only in the short term.

The key implications of this work for an institution are:

1. Firstly, an institution needs a portfolio of interventions to ensure that they can provide the right stimulus for students with different Intention Horizons; and
2. Secondly, an institution needs a portfolio of interventions to encourage progression between Intention Horizons, toward action.

This means that universities may need foster a greater understanding of the journey their students intend to take and thus scope their offerings in different ways; it also suggests that there might be scope to develop highly targeted offerings for specific entrepreneurial needs. For example, Westhead and Solesvik (2016) suggested gender-specific entrepreneurship courses may be advantageous in some cases, and Bell (2019) called for entrepreneurship courses that were aligned to specific departments to enhance entrepreneurial intent. Certainly, it seems true to say that developing the university entrepreneurial ecosystem can result in students with an entrepreneurial mindset, and should lead to graduates with entrepreneurial intent (Isenberg, 2010).

## Conclusion

This research concluded that: firstly, those with intention had a perceived greater need for interventions than those with no intention; secondly, that those with a longer-term view of entrepreneurship were likely to draw on more interventions than those with shorter term intentions; and, finally, that the portfolio of interventions that are perceived as being required are determined, at least to some extent, by the Intention Horizon.

**Fig. 6:**
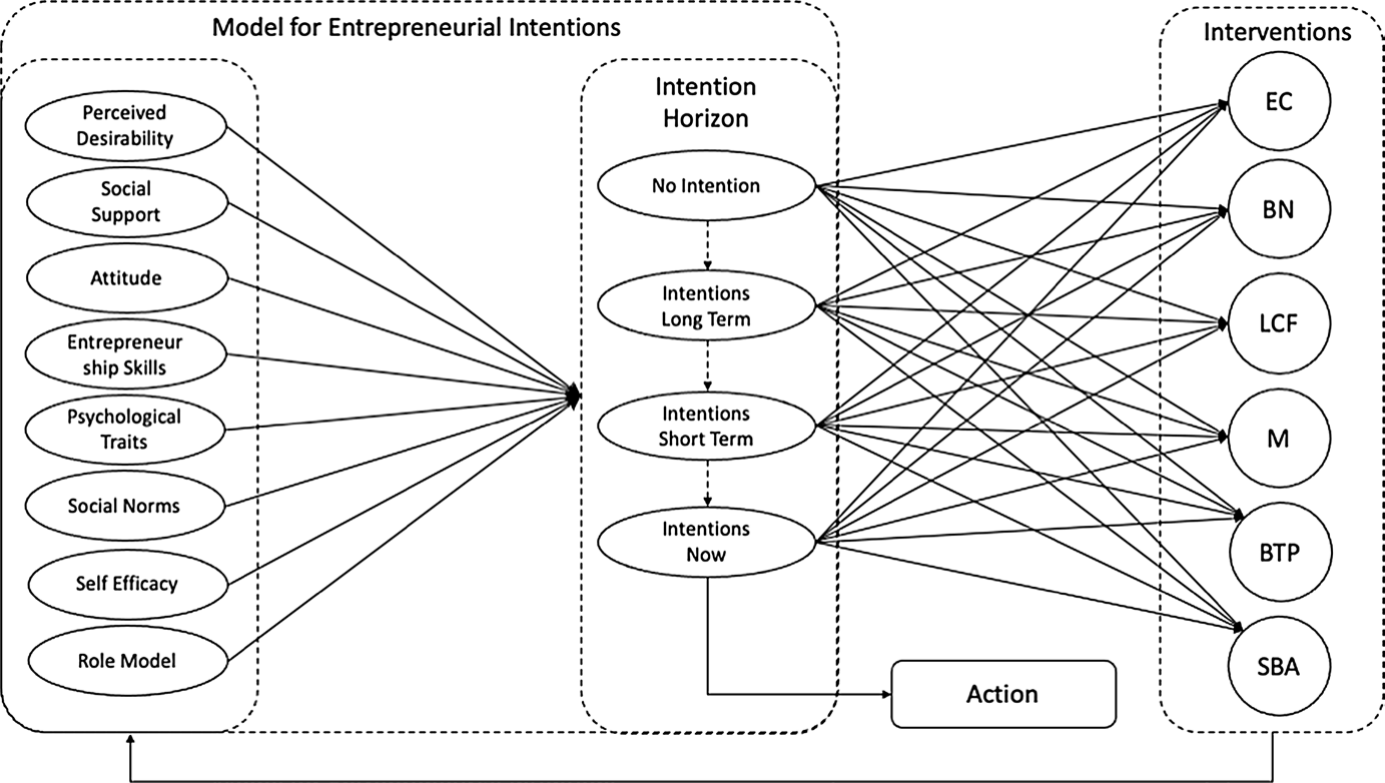
An entrepreneurship education ecosystem model for entrepreneurial intentions

This work also suggests a previously under-articulated relationship between Intention Horizon and interventions which may contribute to resolving some of the debate as to how differing phases of intention can be affected by interventions. The authors of this paper have sought to capture this in Fig. 6.

Herein, it is evident that a range of individuals features shape differing Intention Horizons and these in turn catalyse engagement with interventions.

The works of Fayolle (2005) and more recently Embi et al (2019) and Panwar Seth (2020) have shown that interventions themselves affect the students’ knowledge, skills, values, beliefs, attitude, and support group (the features that impact intention), and this in turn has the potential to affect the nature of their intention, nudging them ever closer to venturing. The authors suggest that this may be an area for further research attention for Intention Horizons.

Practitioners will need to consider these conclusions in the context of their own students' needs and their institutional strategy, and the authors suggest that addition tests of the ideas presented in this study across other cohorts, and exploring additional interventions would be prudent.

That said, staff charged with developing enterprising and entrepreneurial intention in their student body, should pay great heed to the notion that a diverse group of students, such as might be found in any education institution, will likely require a broad offering of interventions to appeal to (and motivate) those with a range of Intention Horizons. Although this approach will likely be the costliest, it should ensure a long-term pipeline of student activity across a range of intervals.

Such an approach should, also, over time, help to develop a supportive ecosystem for students, guiding them to achieve their objectives, and maximising their entrepreneurial potential.

Although it is difficult to make general prescriptions with regard to an institutional offering that might address the broadest number of students, this research suggests that Business Training Programmes may be perceived as being particularly beneficial, followed by Mentoring, Specialist Business Advice, Low-cost Finance, Business Networking Events and Enterprise Clubs.

### Limitations

The authors of this paper were limited in the cohorts available as participants, and the interventions offered by the institutions both present several opportunities to expand upon this work and challenge its central premise.
